# Large variation in the Rubisco kinetics of diatoms reveals diversity among their carbon-concentrating mechanisms

**DOI:** 10.1093/jxb/erw163

**Published:** 2016-04-29

**Authors:** Jodi N. Young, Ana M.C. Heureux, Robert E. Sharwood, Rosalind E.M. Rickaby, François M.M. Morel, Spencer M. Whitney

**Affiliations:** ^1^Department of Geosciences, Princeton University, Princeton, NJ 08544, USA; ^2^Department of Earth Sciences, University of Oxford, South Parks Road, Oxford OX1 3AN, UK; ^3^Plant Science Division, Research School of Biology, The Australian National University, Canberra, ACT 2601, Australia

**Keywords:** Algae, carbon fixation, diatoms, kinetics, photosynthesis, Rubisco.

## Abstract

While marine phytoplankton rival plants in their contribution to global primary productivity, our understanding of their photosynthesis remains rudimentary. In particular, the kinetic diversity of the CO_2_-fixing enzyme, Rubisco, in phytoplankton remains unknown. Here we quantify the maximum rates of carboxylation (*k*_cat_^c^), oxygenation (*k*_cat_^o^), Michaelis constants (*K*_m_) for CO_2_ (*K*_C_) and O_2_ (*K*_O_), and specificity for CO_2_ over O_2_ (S_C/O_) for Form I Rubisco from 11 diatom species. Diatom Rubisco shows greater variation in *K*_C_ (23–68 µM), S_C/O_ (57–116mol mol^−1^), and *K*_O_ (413–2032 µM) relative to plant and algal Rubisco. The broad range of *K*_C_ values mostly exceed those of C_4_ plant Rubisco, suggesting that the strength of the carbon-concentrating mechanism (CCM) in diatoms is more diverse, and more effective than previously predicted. The measured *k*_cat_^c^ for each diatom Rubisco showed less variation (2.1–3.7s^−1^), thus averting the canonical trade-off typically observed between *K*_C_ and *k*_cat_^c^ for plant Form I Rubisco. Uniquely, a negative relationship between *K*_C_ and cellular Rubisco content was found, suggesting variation among diatom species in how they allocate their limited cellular resources between Rubisco synthesis and their CCM. The activation status of Rubisco in each diatom was low, indicating a requirement for Rubisco activase. This work highlights the need to better understand the correlative natural diversity between the Rubisco kinetics and CCM of diatoms and the underpinning mechanistic differences in catalytic chemistry among the Form I Rubisco superfamily.

## Highlight

Broad variations in the CO_2_ fixation kinetics of diatom Rubisco indicate novel mechanistic diversity and large differences in their carbon-concentrating mechanism.

## Introduction

Ribulose-1,5-bisphosphate carboxylase oxygenase (Rubisco) plays a fundamental role in photosynthetic CO_2_ assimilation within the global carbon cycle. Rubisco activity within the terrestrial and ocean biospheres contributes approximately equally to the 10 Pmol of CO_2_ annually fixed into organic carbon (Raven, 2009). Often the rate of CO_2_ fixation is limited by Rubisco activity and, as such, has made the enzyme a primary target to enhance crop photosynthesis and yield through genetic manipulation (Mueller-Cajar and Whitney, 2008; Peterhansel *et al.*, 2008; Carmo-Silva *et al.*, 2015). However, improving Rubisco kinetics has proved difficult as a result of the complex assembly pathway of Rubisco in higher plants (Whitney *et al.*, 2011; Hauser *et al.*, 2015) and apparent trade-offs in its kinetic parameters (Tcherkez *et al.*, 2006; Savir *et al.*, 2010). Widening our understanding of the natural diversity in Rubisco is critical if solutions to improve its performance are to be found and understood (Parry *et al.*, 2013). Of particular interest is Rubisco from organisms adapted to different environments. This includes marine phytoplankton whose efficient carbon-concentrating mechanisms (CCMs) enable them to endure, sometimes thrive in, nutrient- and CO_2_-depleted seawater (Nelson *et al.*, 1995).

In nature, Rubisco is found in a variety of oligomeric forms and within a diverse array of organisms that include archaea, photosynthetic bacteria, cyanobacteria, algae, and plants (Whitney *et al.*, 2011). Form I Rubisco consists of eight large and eight small subunits, and is subdivided into Forms IA–ID depending on its sequence and lineage (Tabita *et al.*, 2008). Most research to date on Rubisco has focused on those sourced from the terrestrial biosphere, with comparatively little characterization of Rubisco from oceanic sources. The terrestrial biosphere is dominated by the ‘green’ chloroplast lineage pertaining to plants and green algae that contain Form IB Rubisco (Tabita *et al.*, 2008). However, oceanic photosynthesis is primarily carried out by phytoplankton containing chloroplasts from a ‘red’ lineage that comprise Form ID Rubisco (Delwiche and Palmer, 1997; Yoon *et al.*, 2002; Falkowski *et al.*, 2004). A group of phytoplankton called diatoms are of particular interest due to their importance in ocean primary productivity (estimated to account for ~20% of global primary production; Nelson *et al.*, 1995), thus influencing global biogeochemical cycles, and for producing silicified walls that preserve paleoclimate signals in the fossil record (Armstrong *et al.*, 2001; Egan *et al.*, 2013; Heureux and Rickaby, 2015).

Compared with other photosynthetic enzymes, Rubisco is considered inefficient due to its low CO_2_-saturated CO_2_ fixation rate (*k*_cat_^c^) and low affinity for CO_2_ (i.e. an elevated Michaelis constant, *K*_m_, for CO_2_; *K*_C_). The basis of this inefficiency arises from the complex catalytic mechanism of Rubisco that imposes biochemical trade-offs between *k*_cat_^c^, *K*_C_, and specificity for CO_2_ over its competitive inhibition by O_2_ (S_C/O_) (Tcherkez *et al.*, 2006). Recent analyses show that the extent of these trade-offs is variable between the Form I Rubisco of plants, red algae, and cyanobacteria (Tcherkez, 2013, 2015). Notably Rubisco oxygenation produces 2-phosphoglycolate, which is toxic to the chloroplast (Zelitch *et al.*, 2009), necessitating its removal via the photorespiratory pathway at a cost of energy and fixed carbon (Peterhansel *et al.*, 2008). The loss of CO_2_ by photorespiration can be as high as 25% of the total carbon fixed in C_3_ flowering plants (Laing *et al.*, 1974).

Despite the catalytic inefficiencies of Rubisco, it appears that it can adapt to the CO_2_:O_2_ ratio of its environment. This is particularly evident for Rubisco kinetics in C_3_ and C_4_ plants. While C_3_ plants rely on diffusion of CO_2_ from the air to chloroplast stroma, C_4_ plants utilize a CCM to elevate CO_2_ around Rubisco to avoid photorespiration and its associated cellular resource costs. In response to higher intracellular CO_2_, C_4_ Rubisco has evolved improvements in *k*_cat_^c^ at the expense of reducing CO_2_ affinity (i.e. increasing *K*_C_) (Yeoh *et al.*, 1980; Seemann *et al.*, 1984; Ghannoum *et al.*, 2005). The CCM and higher *k*_cat_^c^ allow C_4_ plants to reduce their investment in Rubisco, lower the rate of photorespiration, and allow for carbon fixation rates similar to C_3_ plants under low stomatal apertures (Sage, 2004; Ghannoum *et al.*, 2005; Way *et al.*, 2014). These features improve the nitrogen, energy, and water use efficiencies of C_4_ plants.

In the oceans, the low levels of CO_2_, and its slow diffusion rate in water, have led many photosynthetic organisms to evolve CCMs that utilize the higher concentrations of bicarbonate. These mechanisms are different from the CCMs of C_4_ plants that arose during the low CO_2_ concentrations of the Oligocene period (Sage, 2001; Osborne and Sack, 2012) and the coupled warmer, arid environments that trigger stomatal closure and N limitation of the soil (Ehleringer *et al.*, 1997; Long, 1999). The C_4_ plant CCM fixes HCO_3_^−^ by phosphoenolpyruvate (PEP) carboxylase, leading to production of C_4_ organic acids in the Rubisco-lacking mesophyll cells. These C_4_ organic acids then diffuse into the Rubisco-containing bundle sheath cells where they are decarboxylated (Sage, 2004). This process facilitates the concentration of CO_2_ to levels that effectively saturate Rubisco (Furbank and Hatch, 1987).

While some diatoms may also have a C_4_-like mechanism (Reinfelder *et al.*, 2000, 2004) or a C_3_–C_4_ intermediate-like mechanism (Roberts *et al.*, 2007), they contain a CCM suited to their single-celled physiology and high bicarbonate aquatic environment. In diatoms, the CCM consists of various bicarbonate transporters (Nakajima *et al.*, 2013) and differing forms of carbonic anhydrase that serve to elevate CO_2_ levels within the pyrenoid, a low CO_2_-permeable subcellular compartment containing most of the cellular Rubisco (Reinfelder, 2010; Hopkinson *et al.*, 2011). The efficiency of their CCMs allows diatoms to invest their scarce cellular resources conservatively in Rubisco. Accordingly the Rubisco content of diatoms is considerably lower [2–6% (w/w) of total cellular protein] than the 20–50% (w/w) Rubisco content of the soluble protein in plant leaves (Losh *et al.*, 2013; Carmo-Silva *et al.*, 2015).

Our understanding of phytoplankton CCM components and activity regulation remain rudimentary. This is despite the increasing interest in understanding how Form ID Rubisco and CCMs co-evolved and how carbon fixation rates by phytoplankton will respond to rising anthropogenic CO_2_ (Raven *et al.*, 2012; Young *et al.*, 2012, 2013). From the few Form ID Rubisco kinetics determined, there is a strong signal of positive selection within the evolution of the Rubisco large subunits in red algae, Haptophytes, and diatoms (Young *et al.*, 2012). Rubiscos from red algae have the highest specificities for CO_2_ over O_2_ (S_C/O_; ~130–240mol mol^−1^) while the lower S_C/O_ diatom Rubisco (~60–115mol mol^−1^) overlaps with the less diverse S_C/O_ values of C_3_ plant and C_4_ plant Rubisco (~70–90mol mol^−1^) (Read and Tabita, 1994; Whitney *et al.*, 2001, 2011; Haslam *et al.*, 2005). *K*_C_ measurements for diatom Rubisco (~28–40 μM, Badger *et al.*, 1998; Whitney *et al.*, 2001) exceed the concentration of CO_2_ in the surface ocean (~13 μM in air-equilibrated surface seawater at 20 °C), exemplifying the requirement for a CCM. Modeling of the CCM in phytoplankton is highly reliant on measurements of their Rubisco kinetics (Hopkinson, 2011, 2014). However, the paucity of catalysis measurements for diatom Rubisco—and other phytoplankton—continue to limit reliable assessments of CCM function in microalgae.

In this study, we evaluate the diversity of Rubisco kinetics in 11 different diatom species at the common assay temperature of 25 °C. From measurements of S_C/O_, *k*_cat_^c^, *K*_C_, and the *K*_m_ for O_2_ (*K*_O_), we determine the catalytic turnover rate for O_2_ (*k*_cat_^o^) and unveil an unexpected large degree of kinetic variability across the species studied. Uncovered are novel relationships between kinetic parameters not previously observed for other plant and algal Form I Rubisco isoforms. Presented are a novel, robust data set of Rubisco kinetics in marine phytoplankton that provide new insight into potential constraints on microalgal photosynthesis that arise from variations in the effectiveness of their CCM to elevate CO_2_ around Rubisco.

## Materials and methods

### Species selection and sampling:

Eleven species of marine diatoms were selected from cultures maintained at Princeton University and the Australian National University (ANU). Strains from Princeton University were grown at 20 °C under continuous light (~150 μmol photons m^−2^ s^−1^) in 0.2 μm filtered seawater supplemented with Aquil medium (Sunda *et al.*, 2005) and included the diatoms *Thalassiosira weissflogii* (CCMP 1336), *Skeletonema marinoi* (CCMP 1332), *Chaetoceros calcitrans* (CCMP 1315), *Chaetoceros muelleri* (CCMP 1316), and *Phaeodactylum tricornutum* (CCMP 642). *Fragilariopsis cylindrus* (CCMP 1102) was grown at 1 °C, 12:12 light:dark cycle at 75 μmol photons m^−2^ s^−1^. Strains from ANU were grown at 25 °C under a 16:8 light:dark cycle (~150μmol photons m^−2^ s^−1^) cultured in 0.2 μm filtered seawater supplemented with F/2 medium and included the diatoms *Thalassiosisra oceanica* (CS-427), *Chaetoceros calcitrans* (CS-178), *Phaeodactylum tricornutum* (CS-29), *Bellerochea* sp. (CS-874/01), and *Cylindrotheca fusiformis* (CS-13).

### Materials

Unlabeled and ^3^H-labeled ribulose-1,5-bisphosphate (RuBP) was synthesized and purified as described (Kane *et al.*, 1998), and used to prepare ^14^C-labeled carboxypenitol-P_2_ ([^14^C]CPBP) according to Pierce *et al.* (1980) and Zhu and Jensen (1991).

### Rubisco extraction

Cells were harvested at or near exponential growth by gentle centrifugation (2000 *g* for 10min) and the 0.1–0.5ml cell pellets were snap-frozen in liquid nitrogen and stored at –80 °C. Pellets were re-suspended in 5ml of ice-cold extraction buffer containing 50mM EPPS-NaOH, pH 8.0, 1mM EDTA, 2mM DTT, and 1% (v/v) plant protease inhibitor cocktail (Sigma-Aldrich, St Louis, MO, USA), and cells were ruptured in a French press. Extract was centrifuged at 14 000 *g*, 4 °C for 2min and the supernatant was used to quantify Rubisco *K*_C_ *and k*_cat_^c^ or used to purify Rubisco and measure CO_2_/O_2_ specificity (S_C/O_)

### CO_2_/O_2_ specificity

Rubisco was rapidly purified from ~1g (~4–5 pooled biological replicates) of the –80 °C stored cells extracted in ice-cold extraction buffer, and lysed using a French press. Polyvinylpolypyrrolidone [1% (w/v)] was added to the lysate to remove secondary metabolites prior to centrifugation (17 600 *g*, 4 °C, 5min). The soluble cellular protein was rapidly passed over a 1ml Bio-Scale Mini Macro-Prep High Q ion exchange column (Biorad, Hercules, CA, USA) equilibrated with column buffer (50mM EPPS-NaOH, pH 8.0, 1mM EDTA). Bound Rubisco was eluted in 1.5ml of column elution buffer (50mM EPPS-NaOH, pH 8.0, 1mM EDTA 0.8M NaCl) and concentrated to 0.5ml using an Amicon Ultra-15 centrifugal filter (30 000 NMWL, Millipore, Billerica, MA, USA). The protein was applied to the Superdex 200 (GE Life Sciences) column to purify and desalt the Rubisco further. Fractions containing Rubisco were pooled and concentrated again by centrifugal filtration to 0.25ml and glycerol was added to 20% (v/v) final concentration before freezing in liquid nitrogen and storing at –80 °C. Purified Rubisco preparations were used to measure CO_2_/O_2_ specificity using the method of Kane *et al.* (1994).

### *K*_c_, *k*_cat_^c^, *K*_o_, and *k*_cat_^o^

Rubisco content was quantified by [^14^C]2-CABP (2-C-carboxyarabinitol 1,5-bisphosphate) binding as described by Sharwood *et al.* (2008) by incubating duplicate aliquots of soluble cellular protein extracts with 15mM NaHCO_3_, and 15mM MgCl_2_ and either 15 μM or 30 μM [^14^C]CPBP for 10–30min at 25 °C. The recovered amount of [^14^C]CABP-bound Rubisco in both reactions varied by <2% ensuring the Rubisco catalytic site content was accurately quantified. RuBP-dependent ^14^CO_2_ fixation assays were performed in 7ml septum-capped scintillation vials at 25 °C as described (Whitney and Sharwood, 2007) using soluble diatom protein extract following 10–15min activation with 15mM NaH^14^CO_3_ and 15mM MgCl_2_. After activation, kinetic measurements were made on soluble cellular protein in assay vials containing reaction buffer (0.5ml of 100mM EPPS-NaOH, pH 8, 15mM MgCl_2_, 0.6mM ribulose-P_2_, 0.1mg ml^−1^ carbonic anhydrase) equilibrated with 0, 21, 40, and 60% (v/v) O_2_ in N_2_ and five differing concentrations of ^14^CO_2_ (between 10 μM and 100 μM; specific activities of ~1800 cpm nmol^–1^ CO_2_ fixed). The assays were stopped after 1–3min with 0.5 vols of 25% (v/v) formic acid and processed for scintillation counting. Values for *K*_C_ and maximal carboxylase activity (*v*_c_^max^) were extrapolated from the data using the Michaelis–Menten equation as described by Sharwood *et al.* (2008) and Whitney *et al.*, (2011). Measures of *k*_cat_^c^ were calculated by dividing *v*_c_^max^ by the number of Rubisco active sites as determined by [^14^C]2-CABP binding. *K*_O_ was determined from the slope of the linear increase in *K*_C_ in response to O_2_. Values for *k*_cat_^o^ were calculated using the equation *k*_cat_^o^=(*k*_cat_^c^×*K*_O_)/(*K*_C_×S_C/O_). All measurements were made in triplicate using material pooled from 2–3 biological replicates.

### Statistics

One-way ANOVA was performed to determine whether significant differences (*P*-value ≤0.05) existed between the Rubisco kinetics parameters measured in the different groups in Fig. 2. Where significant differences were found, Tukey’s HSD tests were performed to determine which groups differed from each other. Linear regression was fit by least-square analysis, and the correlation coefficient (*r* ^2^) was based on standard error of estimate. Analysis of covariance (ANCOVA) was used to determine whether the linear fit was significant to 95% (*P*-value ≤0.05) in Table 2 and Figs 3 and 4.

## Results

Our results for 11 species of diatoms represent the largest data set of Form ID Rubisco kinetics available to date. The diatom species selected in this study represent a wide range of habitats and evolutionary diversity (Table 1), and included multiple strains of the same species (*P. tricornutum* and *C. calcitrans*), and one polar pennate (*F. cylindrus*).

**Table 1.** Diatom strains, location of isolation, and Rubisco kinetics measured at 25 °C

		*k*_cat_^c^	*K*c	*k*_cat_^o^	*K*_O_	S_C/O_	*K*_*C*_^21%O2^	*k*_*c*at_^c^/*K*_C_	*k*_cat_^c^/*K*_*C*_^21%O2^
**Species**	**Location isolated**	(s^−1^)	(μM)	(s^−1^)	(μM)	(mol mol^−1^)	(μM)	(mM s^−1^)	(mM s^−1^)
**Diatoms (Bacillariophyta**)									
*Thalassiosira weissflogii* (CCMP 1336)^*a*^	Gardiners Island, Long Island, NY, USA	3.2±0.2	65±3	1.3	2032±458	79±1	73	49	44
*Thalassiosira oceania* (CS-427)^*a*^	Little Swan port, Tasmania, Australia	2.4±0.4	65±5	0.4	954±228	80±2	83	36	28
*Skeletonema marinoi* (CCMP 1332)^*a*^	Milford, CT, USA	3.2±1.1	68±8	ND	883±346	ND	88	47	36
*Chaetoceros calcitrans* (CCMP 1315)	Collection site unknown	2.6±0.2	25±1	0.8	413±53	57±11	41	103	64
*Chaetoceros muelleri* (CCMP 1316)	Oceanic Institute, HI, USA	2.4±0.3	23±1.5	0.5	425±67	96±2	37	104	65
*Chaetoceros calcitrans* (CS-178)	Unknown (but should be synonymous with CCMP 1315)	3.4±0.6	31±2	0.7	490±54	75±1	47	112	74
*Bellerochea cf. horologicalis* (CS-874/01)	Ilbilbie, Queensland, Australia	2.1±0.2	50±4	ND	764±190	ND	67	41	31
*Phaeodactylum tricornutum* (UTEX 642)	Plymouth, UK	3.2±0.9	36±1	0.5	592±44	108±2	52	88	62
*Phaeodactylum tricornutum* (CS-29)	Unknown, UK	3.3±0.6	41±1	0.5	664±54	116±2	57	81	59
*Fragilariopsis cylindrus* (CCMP 1102)	Islas Orcadas Cruise, Station 12	3.5±0.3	64±8	0.5	667±255	77±1	88	55	40
*Cylindrotheca fusiformis* (CS-13)	Halifax, Canada	3.7±0.2	ND	ND	ND	79±1	ND	ND	ND
**Higher plants (controls**)									
*Nicotiana tabacum* (tobacco, C_3_)		3.1±0.3	9.7±0.1	1.1	283±15	82±1	18.3	316	167
*Zea mays* (maize, C_4_)		5.5±0.2	19.0±0.6	1.4	397±59	88±2	31	289	177

^*a*^ Belonging to the order Thalassiosirales;

Values shown are average of measurements from *n* ≥3 (± SD) biological repeat samples (see figure legends); ND, not determined

K_C_^21%O2^ was calculated as *K*_C_(1+O_2_/*K*_O_) assuming air-saturated O_2_ levels in H_2_O of 252 µM

### Rubisco activation and stability in diatom cellular protein extract

**Fig. 1.**
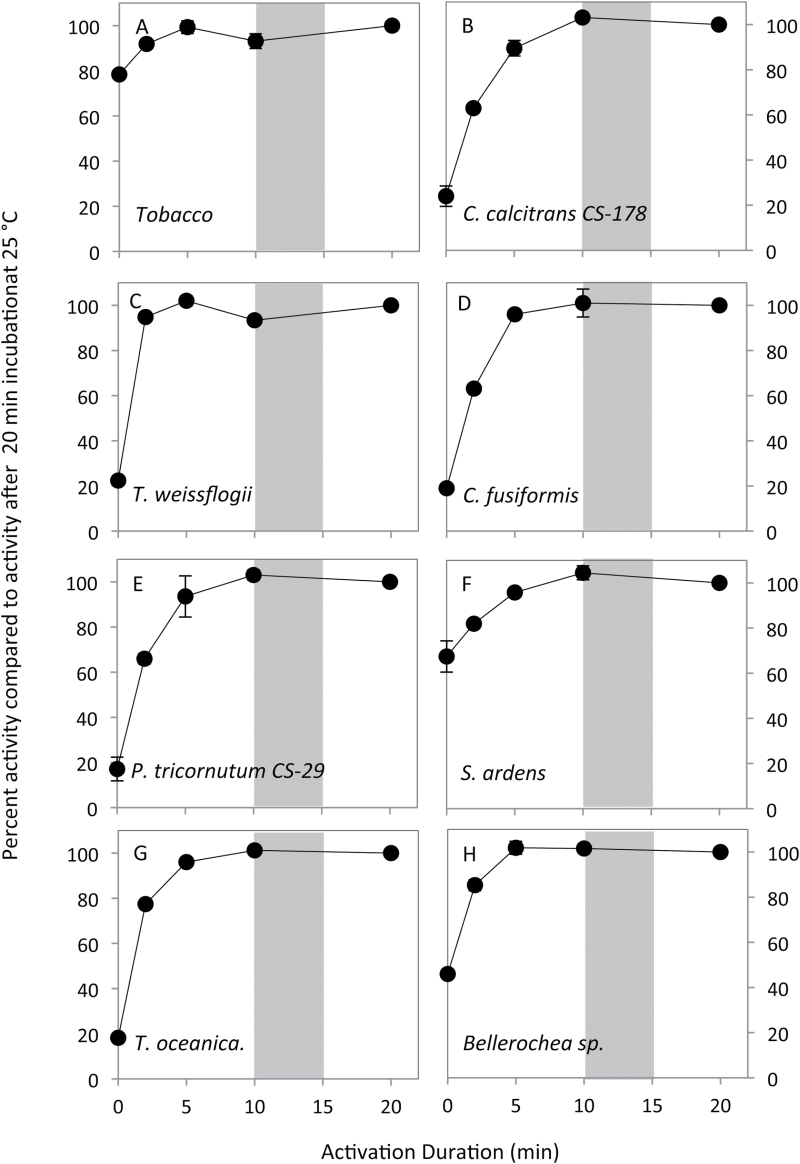
Measurement of Rubisco activation status, maximal activity, and stability *in vitro* at 25 °C. Soluble cellular protein rapidly extracted from tobacco and each phytoplankton in CO_2_-free extraction buffer (containing 5mM MgCl_2_) was used to measure changes in the Rubisco ^14^CO_2_ fixation rate after activating the extract for 0–20min in buffer containing 15mM MgCl_2_ and 15mM NaHCO_3_. Gray shading indicates the time when protein extract was assayed to quantify *k*_cat_^c^, *K*_C_, and *K*_O_ (Table 1). Data represent measures from duplicate biological samples (± SD).

The maintained stability of Rubisco activity in isolated soluble leaf protein facilitates the accurate and reliable measure of maximum carboxylation rates (*k*_cat_^c^) and the Michaelis–Menten (half-saturation) constant (*K*_m_) for carboxylation (*K*_C_) without need of a purification step (Sharwood *et al.*, 2008). These measurements require all eight catalytic sites in each L_8_S_8_ Rubisco molecule to be primed with CO_2_-Mg^2+^ (i.e. activated). *In vivo*, full activity is prevented by inhibitory binding of sugar phosphate molecules to the catalytic site (Andralojc *et al.*, 2012). In illuminated leaves, the ~4:1 molar ratio of RuBP:catalytic sites leads to RuBP binding to non-activated catalytic sites being the almost exclusive cause of inactivation (Price *et al.*, 1995). Upon cellular protein extraction, the levels of available RuBP deteriorate, facilitating RuBP dissociation (and fixation) and catalytic site activation. As shown in Fig. 1A (time zero), Rubisco from newly expanded upper canopy tobacco leaves is ~80% activated *in vivo.* At 25 °C, full activation (i.e. dissociation of all inhibitory RuBP) of tobacco Rubisco in all three leaf samples tested occurred within ~5min *in vitro* and full activity was maintained over the 20min test period.

In contrast to the control tobacco Rubisco *in vitro* activation assays, the activation status of Rubisco in each phytoplankton species was lower, varying between ~20% (*Thalassiosira oceania*, *T. weissflogii*, *Cylindrotheca fusiformis*, and *Phaeodactylum tricornutum*) to ~70% (*Skeletonema ardens*). Accordingly, longer incubation times at 25 °C were required to activate their Rubisco fully *in vitro* (Fig. 1B–H). Nevertheless, in all phytoplankton samples, Rubisco was fully activated within 10min of extraction at 25 °C and the activity was stable for at least a further 10min.

### Rubisco kinetics are highly variable both between and within diatom species.

Measurements of *k*_cat_^c^, *K*_C_, maximum oxygenation rates (*k*_cat_^o^), *K*_m_ for O_2_ (*K*_O_), and the specificity for carboxylase over oxygenase (S_C/O_) were measured at 25 °C to allow for direct comparison with other Rubisco kinetics in the literature. As shown in Table 1, there is significant variation in diatom Rubisco catalysis, with differences even found between Rubisco from the same genus (*Chaetoceros*). Also included in these analyses were control catalysis measurements for Rubisco from tobacco (C_3_ plant) and maize (C_4_ plant) whose values match those previously measured (Supplementary Table S1 at *JXB* online). In the following, we compare our results with kinetics measured at 25 °C for other Form ID Rubiscos from red algae and Form IB Rubisco from C_3_ and C_4_ plants (taken from Badger *et al.*, 1998; Savir *et al.*, 2010).

**Fig. 2.**
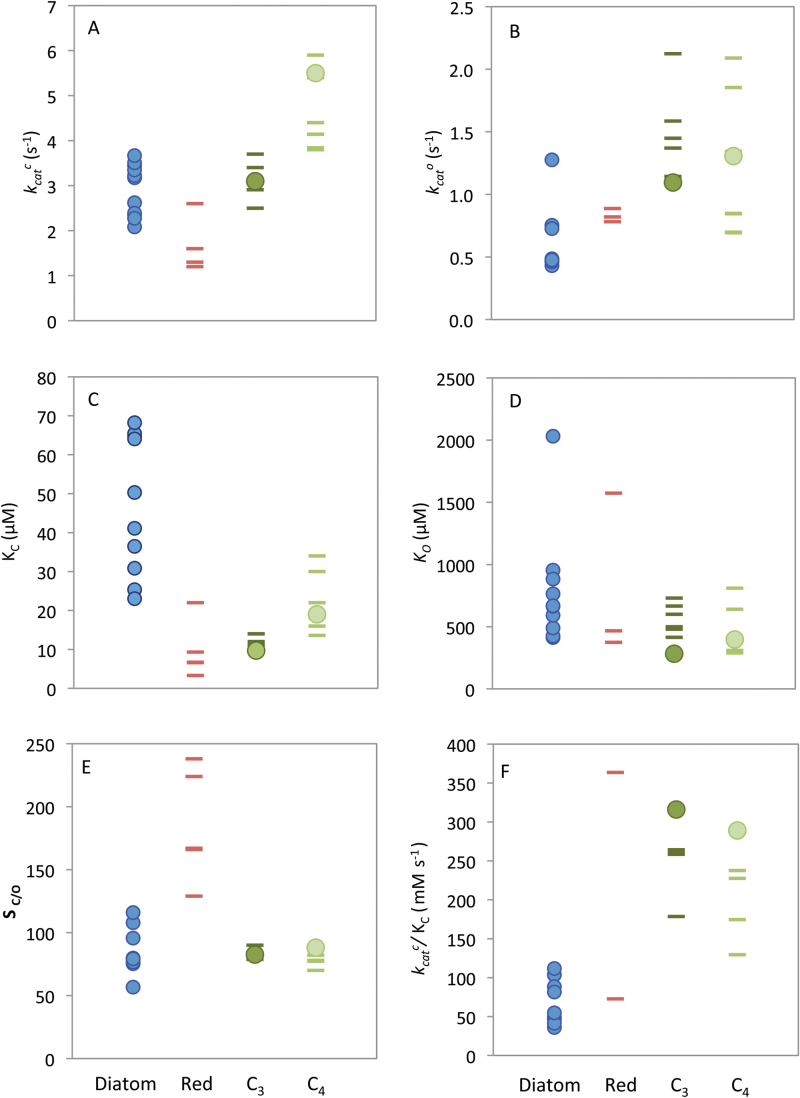
Rubisco kinetic parameters measured at 25 °C. Rubisco properties measured from diatoms (blue circles), tobacco and maize (green circles, see Table 1) compared with previously published values for red algae (red, maroon dashes), C_3_ plants (dark green dashes), and C_4_ plants (light green dashes) (Badger *et al.*, 1998; Savir *et al.*, 2010; Supplementary Table S1). Kinetic parameters include (A) the maximum rates of carboxylation (*k*_cat_^c^), (B) oxygenation rate (*k*_cat_^o^), the *K*_m_ for (C) CO_2_ (*K*_C_) and (D) O_2_ (*K*_O_), (E) the specificity for CO_2_ over O_2_ (S_C/O_), and (F) the carboxylation efficiency (*k*_cat_^c^ */K*_C_, mM s^−1^).

Among the diatom Rubiscos analyzed, there was a <2-fold variation in *k*_cat_^c^, which ranged from 2.1±0.2s^−1^ in *Bellerochea* cf. *horologicalis* to 3.7±0.2s^−1^ in *C. fusiformis*. As shown in Fig. 2A, *k*_cat_^c^ varied significantly between the groups (one-way ANOVA, *F*=25.1, *P*<0.001). Further testing with Tukey HSD showed that diatom *k*_cat_^c^ values were comparable with those of Rubisco from C_3_ plants but statistically lower than those from C_4_ plants (*P*<0.01) and higher than those from red algae (*P*<0.01). Diatom Rubisco also showed diversity in the oxygenation rates (Fig. 2B). The measured *k*_cat_^o^ values of 0.4–1.6s^−1^ were significantly lower than those of C_3_ plants, but not lower than those of C_4_ plants or red algae (one-way ANOVA, *F*=5.25, *P*=0.008, Tukey HSD between diatoms and C_3_ plants *P*=0.007).

With regard to CO_2_ affinity, the *K*_C_ values of diatom Rubisco varied >2.7 fold (25–68 μM; Fig. 2C) and were significantly higher than those of Rubisco from red algae, and C_3_ and C_4_ plants (one-way ANOVA, *F*=19.5, *P*<0.001, Tukey HSD *P*<0.01 for all three pairs). Only cyanobacteria have a lower CO_2_ affinitty than diatoms, with *K*_C_ values in the range 200–260 μM, which accords with the effectiveness of their CCM (Price *et al.*, 2008; Whitney *et al.*, 2011; Hopkinson *et al.*, 2014). In contrast, diatom Rubisco *K*_O_ values were not statistically different from those of red algae, C_3_, or C_4_ plants (one-way ANOVA, *F*=1.44, *P*=0.26). Of particular interest was the low O_2_ affinity of *T. weissflogii* Rubisco whose *K*_O_ exceeded 2mM O_2_.

Improving the S_C/O_ of Rubisco, without unfavorably changing its other kinetic parameters, is a prized goal as it has a pervasive influence on Rubisco efficiency in organisms both with and without CCMs (Long *et al.*, 2015). While the S_C/O_ range in C_3_ and C_4_ plants shows limited diversity, a 2-fold variation was found in the S_C/O_ of diatom Rubisco at 25 °C (i.e. 57–116mol mol^−1^; Table 1) and encompassed the range found previously in diatoms (Badger *et al.*, 1998; Whitney *et al.*, 2001; Haslam *et al.*, 2005). Despite this large variation, the S_C/O_ of diatom Rubisco was not significantly different from that of C_3_ and C_4_ plants, and failed to reach the high S_C/O_ of Rubisco from red algae (Fig. 2E). In terms of carboxylation efficiency (*k*_cat_^c^ */K*_C_), we find that diatom Rubisco is low compared with red algae, and C_3_ and C_4_ plants (one-way ANOVA, *F*=11.5, *P*=0.0002, Fig. 2F, Tukey HSD *P*=0.04, *P*=0.001, and *P*=0.005, respectively), even in the presence of ambient O_2_ levels (i.e. *k*_cat_^c^/*K*_C_^21%O2^; Table 1).

### Novel kinetic relationships of diatom Rubisco

A number of trade-offs between Rubisco kinetic parameters have been observed across a range of primary producers (Tcherkez *et al.*, 2006; Savir *et al.*, 2010). From the relatively small data set examined, relationships between the varied Rubisco kinetic parameters (i.e. *K*_C_, *k*_cat_^c^, *k*_cat_^o^, S_C/O_, and *K*_O_) appear confined to a one-dimensional landscape, with simple power law correlations between parameters (Savir *et al.*, 2010). We examined these relationships by comparing the kinetic parameters of diatom Rubisco with those measured in green algae, red algae, and plants using data sets of Tcherkez *et al.* (2006), Savir *et al.* (2010), Whitney *et al.* (2011), and Galmes *et al.* (2014) (see Supplementary Table S1).

**Fig. 3.**
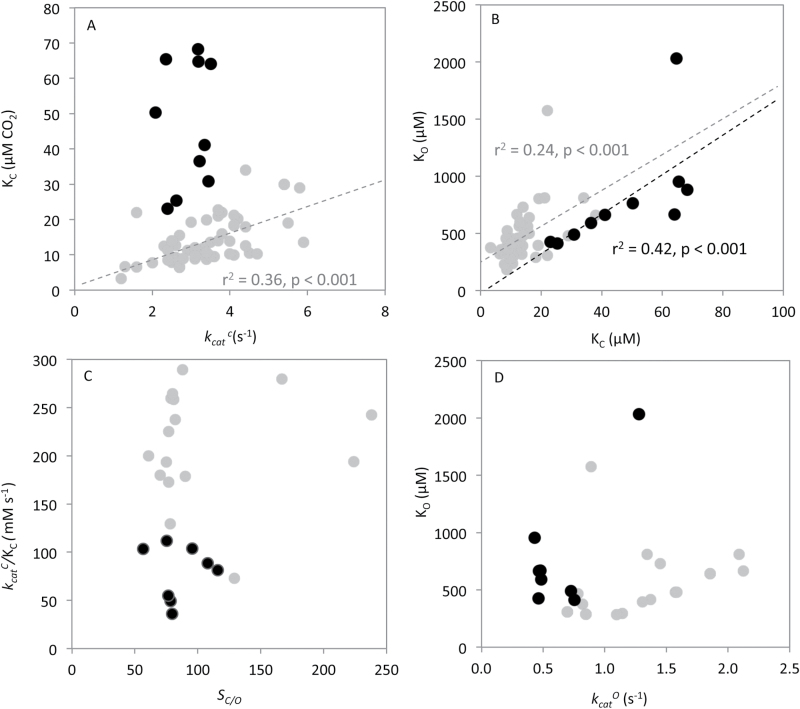
Comparing the catalytic relationships of diatom and other Form I Rubiscos. Comparison of (A) maximum carboxylation rate (*k*_cat_^c^) versus the *K*_m_ for CO_2_ (*K*_C_), (B) *K*_C_ versus the *K*_m_ for O_2_ (*K*_O_), (C) the carboxylation efficiency (*k*_cat_^c^/*K*_C_) versus specificity for CO_2_ over O_2_ (S_C/O_), and (D) the maximum oxygenation rate (*k*_cat_^o^) and *K*_O_. Measured for diatoms (black circles) and compared with the compilation of plants and eukaryotic algae from Savir *et al.* (2010), Badger *et al.* (1998), Whitney *et al.* (2011), and Galmes *et al.* (2014) (gray circles).

As shown recently by Tcherkez (2015), the well-documented trade-off between *K*_C_ and *k*_cat_^c^ varies between organisms. For plant and algal Form I Rubisco, the correlation between *K*_C_ and *k*_cat_^c^ varies from that seen for Form I Rubisco from cyanobacteria and other prokaryotes (Tcherkez, 2013). With regard to eukaryotic Form I Rubisco, the diatom variants uniquely show no correlation between *K*_C_ and *k*_cat_^c^ (Fig. 3A). The linear correlation of *r* ^2^=0.36, *P*=1.1×10^–6^ between *k*_cat_^c^ versus *K*_C_ for plant and eukaryotic algal Form I Rubisco (Fig. 3A, gray circles), became insignificant when diatom data were included (Fig. 3A, black circles, *r* ^2^=0.013, *P*=0.36). This suggests that the catalytic mechanism of diatom Rubisco may differ relative to other eukaryotic variants—possibly through changes to one or more of the elemental steps in the Rubisco catalytic cycle.

A positive linear correlation between *K*_C_ and *K*_O_ was found among diatom Rubisco (*r* ^2^=0.43, *P*=0.041; Fig 3B) that was strengthened when the outlying high *K*_O_ value (i.e. low O_2_ affinity) for *T. weissflogii* Rubisco was removed (*r* ^2^=0.83, *P*=5.8×10^–4^). Form 1 Rubisco from other plants and eukaryotic algae also displays a significant positive correlation (*r* ^2^=0.24, *P*=3.73×10^–4^), and the strength of this relationship increases (*r*=0.41, *P*=5.0×10^–8^) when the diatom measurements are included.

Diatom Rubiscos also showed no statistically significant relationship between carboxylase efficiencies and S_C/O_ (*r* ^2^<0.002, *P*=0.5; Fig 3C). Although diatom carboxylation efficiencies are lower than those of higher plant Rubisco (Fig 2F), the diatom S_C/O_ values are within a similar range, indicating that oxygenase efficiencies must also be correspondingly lower than those of plants, probably due to the lower *k*_cat_^o^ rates of diatom Rubisco (Fig 2B).

While a weak positive relationship between *k*_cat_^o^ and *K*_O_ has been shown by Tcherkez (2015), we found that such a correlation did not extend to diatom Rubisco (*r* ^2^=0.38, *P* =0.078) (Fig. 3D, black circles) or when combined with plant and other eukaryotic algal Rubisco (Fig. 3D, gray circles). In addition, no relationship between *k*_cat_^c^ and either *K*_C_/*K*_O_ or S_C/O_ was evident in the diatom Rubisco data set, although for these comparisons our values fall within the noise of the data analyzed by Savir *et al.* (2010). Linear regressions were tested between all kinetic parameters of diatom Rubiscos and are shown in Table 2.

**Table 2.** Linear correlations between various Rubisco kinetic parameters from 11 diatom species

	*k*_cat_^c^	*K*_C_	*K*_O_^*a*^	S_C/O_	*k*_cat_^o^	*k*_cat_^c^/*K*_C_	*k*_cat_^o^/*K*_O_	*K*_C_ */K*_O_	*K*_C_^21%O2^	*k*_cat_^*c*^/*K*_C_^21%O2^
*k*_cat_^c^	1	0.0212 (0.688)	0.0133 (0.752)	0.0182 (0.729)	0.0503 (0.562)	0.0388 (0.585)	0.0016 (0.919)	0.0299 (0.633)	0.0293 (0.637)	0.103 (0.365)
*K*_C_		1	**0.425 (0.0412**)	0.00965 (0.817)	0.181 (0.254)	**0.854 (0.000131**)	0.223 (0.199)	0.0715 (0.455)	**0.958 (<0.00001**)	**0.760 (0.00102**)
*K*_O_^*a*^			1	0.00818 (0.831)	0.378 (0.0783)	0.350 (0.0714)	0.209 (0.216)	0.275 (0.120)	0.240 (0.150)	0.217 (0.175)
S_C/O_				1	0.178 (0.298)	0.00189 (0.919)	**0.260 (0.196**)	0.00782 (0.835)	0.0126 (0.791)	0.00195 (0.742)
*k*_cat_^o^					1	0.107 (0.390)	0.125 (0.351)	0.0914 (0.429)	0.108 (0.389)	0.0667 (0.502)
*k*_cat_^c^/*K*_C_						1	0.304 (0.124)	0.0425 (0.568)	**0.803 (0.000448**)	**0.963 (<0.00001**)
*k*_cat_^o^/*K*_O_							1	0.00552 (0.849)	0.176 (0.260)	0.228 (0.194)
*K*_C_/*K*_O_								1	0.210 (0.183)	0.0888 (0.403)
*K*_C_ air									1	**0.752 (0.0116**)
*k*_cat_^c^/*K*_C_ air										1

Correlation coefficient and probability of linear correlation, *r* ^2^ (*P*-value).

Relationships with statistically significant linear correlation (*P*<0.05) are shown in bold.

^*a*^ Correlation when all data are included. This is different from the correlation shown in Fig. 2, which does not include the T. *weissflogii* outlier.

### Rubisco carboxylation efficiency versus Rubisco content

**Fig. 4.**
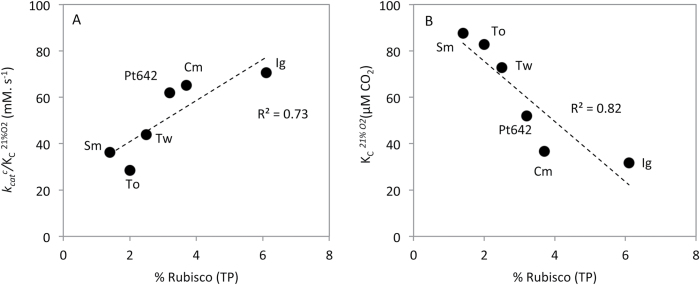
Relationships of Rubisco content to catalysis. (A) Rubisco content as a percentage of total cellular protein (% TP) is positively correlated with carboxylation efficiency in 21% O_2_ (*k*_cat_^c^/*K*_C_^21%O2^). (B) This is largely driven by the negative correlation of Rubisco content with *K*_C_^21%O2^. Rubisco content was taken from Losh *et al.* (2013). *I. galbana* (Ig), *C. muelleri* (Cm), *P. tricornutum* 642 (Pt642), *T. weissflogii* (Tw), *T. oceanica* (To), and *S. marinoi* (Sm).

Compared with C_3_ plants, the faster carboxylation rates of Rubisco in C_4_ plants enable them to invest less of their N resources in Rubisco (Seemann *et al.*, 1984; Ghannoum *et al.*, 2005). We therefore compared the Rubisco content measured in a range of diatoms and the Haptophyte *Isochrysis galbana* in exponentially growing cells (same strains grown under the same conditions; see Losh *et al.*, 2013) against our measured values of *k*_cat_^c^ and *K*_C_ (Table 1). We found no relationship between Rubisco content and *k*_cat_^c^, but observed a positive correlation between Rubisco content and carboxylation efficiency in the presence of 21% O_2_ (*k*_cat_^c^/*K*_C_^21%O2^), which is driven by the negative correlation of Rubisco content with *K*_C_^21%O2^ (Fig. 4). These relationships are also apparent when Rubisco content is plotted against carboxylation efficiency and *K*_C_ in the absence of O_2_, but with a lower correlation coefficient.

## Discussion

In this study, we demonstrate the large catalytic diversity of Rubisco among 11 diatom species. A unique property of diatom Rubisco is that it lacks the relationship between CO_2_-fixing speed (*k*_cat_^c^) and CO_2_ affinity (*K*_C_) shared by many higher plant and algal Form I Rubisco isoforms. Our measured K_C_ values for diatom Rubisco show larger diversity than observed in contemporary plant Form I Rubisco and, as a result, would require different CO_2_ concentrations to saturate Rubisco. Our data suggest that the current estimates of CO_2_ concentrations at the site of Rubisco in diatoms are significantly underestimated for some species. In addition, there is an unexpected negative relationship between *K*_C_^21%O2^ and the Rubisco content in diatoms that suggests a trade-off in the allocation of resources (energy and nutrients) between investing in Rubisco production to sustain rates of photosynthesis for competitive growth or enhancing CCM capacity to sustain saturating CO_2_ levels for Rubisco function.

### No significant relationship exists between *k*_cat_^c^ and *K*_C_

A positive relationship between *k*_cat_^c^ and *K*_C_ has been found in all Rubiscos studied to date, although there are increasing indications from wider surveys of Rubisco kinetics (Galmes *et al.*, 2014) that the relationship shared by plant and algal Rubisco differs from that observed for prokaryotic Form I Rubisco (Tcherkez, 2013, 2015). Remarkably, diatom Rubiscos in this study show no relationship between *K*_C_ and *k*_cat_^c^, whereby their C_3_ plant-like *k*_cat_^c^ values (Fig. 2A) contrast with atypically high *K*_C_ values that generally exceed those of C_4_ plant Rubisco (Fig. 2C). The lack of relationship is surprising as the trade-off between *K*_C_ and *k*_cat_^c^ is thought to be due to a fundamental mechanistic constraint of their inter-related rate constants (Tcherkez *et al.*, 2006, 2012). However, differences in the relationships of *k*_cat_^c^ and *K*_C_ between different photosynthetic groups may arise from differences in the intrinsic equilibrium of the RuBP enolization reaction (Tcherkez, 2013, 2015). More study is needed into the enolization, CO_2_/O_2_ addition, hydration, and cleavage reactions of Rubisco from diatoms (and other microalgae) to understand fully the extent and mechanistic foundation for the contrasting relationships between *k*_cat_^c^ and *K*_C_ found in nature.

### High *K*_C_ values require higher concentrations of CO_2_ to saturate

Diatom Rubisco *K*_C_ values are significantly higher than the concentration of CO_2_ in seawater (~10 μM at 25 °C). Like C_4_ plants, cyanobacteria, and many other photosynthetic organisms, diatoms are rarely limited by CO_2_ for growth because they possess a CCM. The CCM varies between species, but all combine both morphological (such as the pyrenoid, carboxysome, and bundle sheath in diatoms, cyanobacteria, and C_4_ plants, respectively) and biochemical (e.g. carbonic anhydrases and bicarbonate transporters) specialization to provide high CO_2_ concentrations around the site of Rubisco. In C_4_ plants, CO_2_ concentrations within the bundle sheath chloroplasts are in excess of 160 μM (i.e. >5000 μbar; Furbank and Hatch, 1987) relative to the subsaturating CO_2_ concentrations in C_3_ chloroplasts (<10 μM). Similarly, the CCM of cyanobacteria provides highly saturating CO_2_ levels of >400 μM within the carboxysome for their Rubisco (assuming a pH of 7.35 and a 15mM inorganic carbon pool; Hopkinson *et al.*, 2014; Whitehead *et al.*, 2014).

Experimental determination of concentrations of CO_2_ and O_2_ in pyrenoids of diatoms is currently impossible. This has necessitated CO_2_ levels being modeled according to conceptual understanding of the diatom CCM and its Rubisco kinetics. Based on a *K*_C_^21%O2^ of ~41 μM for *P. tricornutum* Rubisco (Whitney *et al.*, 2001), a pyrenoid CO_2_ concentration of ~110 μM was modeled (Hopkinson, 2014). While only 9-fold higher than surface seawater CO_2_ concentrations, this CO_2_ level would give ~75% saturation of Rubisco carboxylase activity. As shown in Table 1, this assumed *K*_C_^21%O2^ value is at the lower end of the values measured in our study, indicating that most diatom species would require higher concentrations of CO_2_ in the pyrenoid to attain similar saturation. For example, from our measures of *K*_C_^21%O2^ (37–88 μM) the pyrenoid CO_2_ concentrations would need to range between ~148 μM and 352 μM for 80% saturation (or ~92–272 μM in the absence of O_2_). This adjustment would suggest that CO_2_ concentrations in the pyrenoid are similar to, and possibly exceed, those measured in C_4_ plant bundle sheath cells; see above and Furbank and Hatch (1987).

### Energetic investment in the CCM and Rubisco

The single-cell physiology of phytoplankton and variable nutrient availability of marine ecosystems require their expedient use and probably restricts the energy and resources that can be expended on photosynthesis. The investment of cellular resources in Rubisco synthesis is probably limited to the minimum concentration required for growth (Losh *et al.*, 2013) albeit in balance with the requirements of other cellular processes, such as the high energy costs of a CCM. Notably the resource investment by diatoms in Rubisco [e.g. 2–6% (w/w) of cellular protein; Fig. 3] is much smaller than that in the leaf soluble protein of C_3_ plants [25–50% (w/w)] and C_4_ plants [10–40% (w/w)] where it accounts for 5–25% of leaf N (Ghannoum *et al.*, 2005; Carmo-Silva *et al.*, 2015). The energy cost of a CCM depends on the capacity of phytoplankton to actively take up inorganic carbon at a rate that is proportional to the diffusive loss of CO_2_ from the cell, which is influenced by the internal:external CO_2_ gradient and the permeability of the pyrenoid to CO_2_ (Hopkinson *et al.*, 2011; Raven *et al.*, 2014; Raven and Beardall, 2016). Diatoms appear well adapted to maintaining near-saturating CO_2_ levels around Rubisco (Tortell *et al.*, 2000; Rost *et al.*, 2003; Chen and Gao, 2004; Kranz *et al.*, 2015) by regulating CCM activity in response to varying extracellular CO_2_ (Chen and Gao, 2004; Hennon *et al.*, 2015; Young *et al.*, 2015). This ‘energy minimization’ strategy in CCM regulation and Rubisco production probably provides an N minimization strategy and a significant growth advantage to diatoms (Giordano *et al.*, 2005; Raven *et al.*, 2011).

**Fig. 5.**
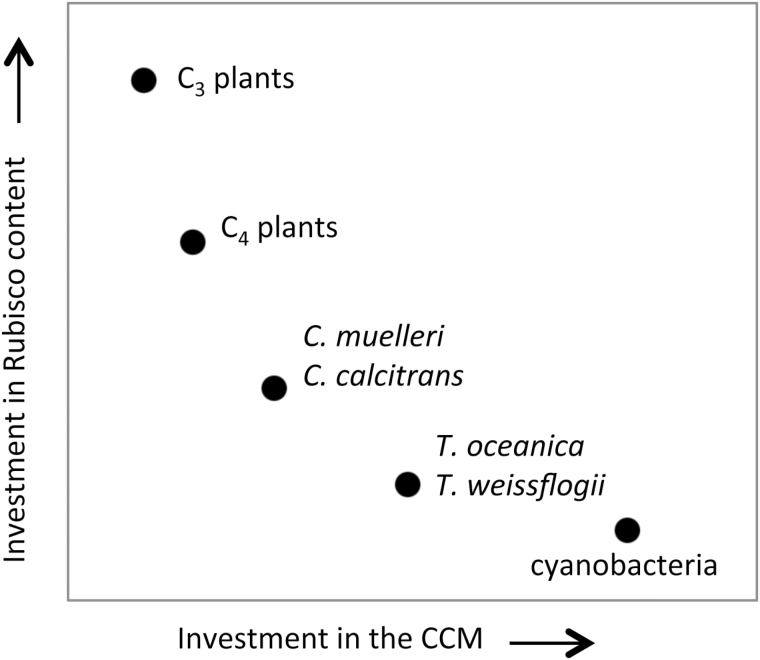
Resource allocation hypothesis for the balance between Rubisco content and *K*_C_ values. Organisms that invest their energy resources heavily in the CCM (e.g. cyanobacteria) to maintain high intracellular CO_2_ levels to saturate Rubisco and limit photorespiration are able to reduce their resource investment in Rubisco. At the other end of the spectrum, organisms without a CCM (e.g. C_3_ plants) have Rubisco that is undersaturated with CO_2_ and require large resource investments in Rubisco content to maintain adequate rates of carbon fixation for survival. Organisms with a CCM fall somewhere along the saturation curve, depending on the carboxylation speed of their Rubisco and their potential to balance the investment of resources in Rubisco biogenesis suitably or maintain elevated intercellular CO_2_ levels around Rubisco.

Our study provides evidence for linkages between Rubisco CO_2_ affinity and the efficiency of the CCMs in diatoms. Unlike in C_4_ plants where improvements in *k*_cat_^c^ correlate with reduced N investment in Rubisco (Ghannoum *et al.*, 2005), diatom Rubisco content was positively correlated with carboxylation efficiency (Fig. 4A) not *k*_cat_^c^. This correlation was governed primarily by the strong negative relationship between Rubisco content and *K*_C_ (both with and without 21% O_2_; Fig. 4B). This leads us to hypothesize that diatoms balance resource allocation for photosynthesis between Rubisco content or the CCM. Diatoms such as *Thalassiosira* and *Skeletonema* species maintain low Rubisco content but require more resource allocation to their CCM to saturate their low-CO_2_ affinity (high *K*_C_) Rubiscos. Alternatively, diatoms such as *Phaeodactylum* and *Chaetoceros* species have higher Rubisco content and lower *K*_C_ values, requiring lower CO_2_ concentrations for saturation of carboxylation (Fig. 4). As summarized in Fig. 5, this hypothesis infers an evolutionary trade-off between Rubisco kinetics and content in diatoms; that is, the level of energy invested by diatoms in their CCM to attain CO_2_ concentrations suited to their Rubisco CO_2_ affinity, not its *k*_cat_^c^, influences resource availability for cellular metabolism, that includes Rubisco synthesis.

### Co-evolution of Rubisco and CCMs

Variations in the mechanistic chemistry of diatom Rubisco and plasticity in CCM efficiency among diatom species may explain the non-canonical kinetic features identified for diatom Rubisco (Fig. 3). Although the common ancestor of diatoms was thought to have gained a chloroplast from secondary endosymbiosis of a red alga ~1.2 billion years ago (Yoon *et al.*, 2002), diatoms only began to appear in the fossil record ~200 million years ago (Brown and Sorhannus, 2010). When, or how, the subsequent falling CO_2_:O_2_ levels triggered the evolution of marine CCMs remains uncertain (Raven *et al.*, 2012). Notably, although sharing similar Form ID Rubiscos, the S_C/O_ and carboxylation efficiencies of red algae are much higher than those of diatoms and, in some cases, those of higher plants (Whitney *et al.*, 2001). Comparative analyses of the large subunit for a small number of Form ID Rubiscos showed a clear signal of positive selection both between and within the major algal groups (Young *et al.*, 2012). In particular, positive selection was detected along the basal branch leading to diatoms, including between the centric diatom Thalassiosirales and Chaetocerales species, which accords with the very different *K*_C_ values we observed in this study. Further work is needed to understand adaptation of Form ID Rubisco and to elucidate the amino acid changes in the large and/or small subunits responsible for the wide range of kinetic parameters observed in this study.

### A requirement for Rubisco activase in diatoms

A unique outcome of this study was the finding of the low and variable activation status of Rubisco in diatoms (~20–70%, Fig. 1) that implies a requirement for accessory factors for functional maintenance. As in higher plants, deactivation of diatom Rubisco probably results from loss of Mg^2+^ and the carbamyl prosthetic group in the catalytic site, allowing for autoinhibition by substrate RuBP binding (to form ‘ER’ complexes). Likewise, binding of other inhibitory sugar phosphate molecules might occur to fully activated Rubisco (Andralojc *et al.*, 2012). Removal of the sugar phosphate is facilitated by Rubisco activase (RCA) via conformational remodeling driven by ATP hydrolysis (Mueller-Cajar *et al.*, 2014). In nature, differing types of RCA have independently evolved among plants, non-green algae (called CbbX; Mueller-Cajar *et al.*, 2013), and photosynthetic prokaryotes (called CbbOQ; Tsai *et al.*, 2016). While an RCA function in diatoms has not been demonstrated, they encode a chloroplast and nuclear *cbb*X gene (Kroth, 2015), consistent with our findings of a need for Rubisco activity regulation. The low activation status of diatom Rubisco somewhat parallels the low Rubisco activation status in C_4_ plants (~40–60%; von Caemmerer and Furbank, 2003) and in C_3_ plants grown at high CO_2_ (e.g. it is <50% in tobacco grown in air containing 0.3% v/v CO_2_; Whitney *et al.*, 1999). Understanding how the regulatory properties of diatom RCA and the activation status of Rubisco differ in response to environmental conditions (e.g. temperature, illumination, CO_2_, and nutrients) and impact the resource use efficiency of diatoms have yet to be examined.

### Photorespiration in diatoms

Analogous to other Form I Rubisco (Savir *et al.*, 2010; Galmes *et al.*, 2014), diatom Form ID Rubisco showed a positive relationship between *K*_C_ and *K*_O_ (Fig. 3B). This indicates that unwanted reductions in CO_2_ affinity (i.e. increasing *K*_C_) are complemented by favorable reductions in O_2_ affinity (i.e. increasing *K*_O_). These kinetics help to ensure that the rates of photorespiration are not exacerbated in diatoms. The corresponding effect of specificity for CO_2_ over O_2_ (S_C/O_) is quite variable, spanning values that match or exceed the S_C/O_ of C_3_ and C_4_ plant Rubisco (Fig. 2E). As highlighted by Whitney *et al.* (2001), the lower carboxylation efficiencies under 21% O_2_ (*k*_cat_^c^/*K*_C_^21%O2^) shared by all diatom Rubiscos relative to plant Rubisco (Table 1) precludes them from being more efficient enzymes within the context of a C_3_ plant chloroplast—irrespective of the higher S_C/O_ values measured for some diatom Rubisco variants.

As in other CCM-containing organisms, the rates of photorespiration in diatoms will be suppressed under the higher CO_2_ concentrations maintained around Rubisco in the pyrenoid. To date, however, little is known about the photorespiration process and rate in diatoms (Schnitzler Parker *et al.*, 2004; Rigobello-Masini *et al.*, 2012). As indicated above, resolving our understanding of diatom CCMs, and the O_2_ levels in the pyrenoid, is vital to assessing the susceptibility of diatoms to photorespiration. One interpretation from the wide range in *K*_O_ and S_C/O_ values measured between the diatom species is that the CO_2_:O_2_ pressures within the pyrenoid may differ dramatically. This may arise through variation in the effectiveness of their CCM to concentrate CO_2_ or through reduced permeability to O_2_, or both. Particulaly curious is the elevated O_2_ insensitivity (i.e. high *K*_O_ value) of Rubisco from the centric diatom *T. weissflogii*. This suggests that there may be different biochemical constraints on the Rubisco in this species worthy of further study.

### Conclusions

Primary production in the oceans is dominated by phytoplankton, with diatoms accounting for ~20% of global primary production (Falkowski and Raven, 2007). Here we provide the largest data set of diatom Rubisco kinetics that identifies large catalytic diversity and novel relationships in their properties. The data suggest that the CCM of diatoms is highly diverse and capable of concentrating CO_2_ in the pyrenoid to higher levels than currently envisaged. Our findings also suggest that the CO_2_ affinity of diatom Rubisco is a key indicator for how these microalgae manage the allocation of their relatively scarce cellular resources between Rubisco biogenesis and components of their CCM. This work highlights the importance of further studying the phylogenetically diverse, non-terrestrial, Rubisco isoforms to decipher potential mechanistic differences in the catalytic chemistry of the Form I Rubisco superfamily.

## Supplementary data

Supplementary data are available at *JXB* online.

Table S1. Rubisco kinetics at 25 °C taken from other data sets and used in Fig. 2

Supplementary Data

## References

[CIT0001] Andralojc PJ Madgwick PJ Tao Y 2012 2-Carboxy-d-arabinitol 1-phosphate (CA1P) phosphatase: evidence for a wider role in plant Rubisco regulation. Biochemical Journal 442, 733–742. 22132794 10.1042/BJ20111443

[CIT0002] Armstrong RA Lee C Hedges JI Honjo S Wakeham SG 2001 A new, mechanistic model for organic carbon fluxes in the ocean based on the quantitative association of POC with ballast minerals. Deep Sea Research Part II: Topical Studies in Oceanography 49, 219–236.

[CIT0003] Badger MRT Andrews J Whitney SM Ludwig M Yellowlees DC Leggat W Price GD 1998 The diversity and coevolution of Rubisco, plastids, pyrenoids, and chloroplast-based CO_2_-concentrating mechanisms in algae. Canadian Journal of Botany 76, 1052–1071.

[CIT0052] Brown, JW. and Sorhannus, U. (2010) A molecular genetic timescale for the diversification of autotrophic Stramenophiles (Ochrophyta): Substantive underestimation of putative fossil ages. PLoS ONE 5(9):e12759. 20862282 10.1371/journal.pone.0012759 PMC2940848

[CIT0004] Carmo-Silva E Scales JC Madgwick PJ Parry MAJ 2015 Optimizing Rubisco and its regulation for greater resource use efficiency. Plant, Cell and Environment 38, 1817–1832. 10.1111/pce.12425 25123951

[CIT0005] Chen X Gao K 2004 Photosynthetic utilisation of inorganic carbon and its regulation in the marine diatom *Skeletonema costatum* . Functional Plant Biology 31, 1027–1033. 10.1071/FP04076 32688971

[CIT0006] Delwiche CF Palmer JD 1997 The origin of plastids and their spread via secondary symbiosis. In: Bhattacharya D, ed. The origins of algae and their plastids. Vienna: Springer-Verlag, 53–86.

[CIT0007] Egan KE Rickaby REM Hendry KR Halliday AN 2013 Opening the gateways for diatoms primes Earth for Antarctic glaciation. Earth and Planetary Science Letters 375, 34–43.

[CIT0008] Ehleringer JR Cerling TE Helliker BR 1997 C_4_ photosynthesis, atmospheric CO_2_, and climate. Oecologia 112, 285–299. 10.1007/s004420050311 28307475

[CIT0009] Falkowski P Schofield O Katz M Van de Schootbrugge B Knoll A 2004 Why is the land green and the ocean red? In: Thierstein H Young J, eds. Coccolithophores. Berlin: Springer, 429–453.

[CIT0010] Falkowski PG Raven JA 2007 Aquatic photosynthesis, Vol. 2 Princeton, NJ: Princeton University Press.

[CIT0011] Furbank RT Hatch MD 1987 Mechanism of C_4_ photosynthesis: the size and composition of the inorganic carbon pool in bundle sheath cells. Plant Physiology 85, 958–964. 16665838 10.1104/pp.85.4.958 PMC1054376

[CIT0012] Galmes J Kapralov MV Andralojc PJ Conesa MÀ Keys AJ Parry MAJ Flexas J 2014 Expanding knowledge of the Rubisco kinetics variability in plant species: environmental and evolutionary trends. Plant, Cell and Environment 37, 1989–2001. 10.1111/pce.12335 24689692

[CIT0013] Ghannoum O Evans JR Chow WS Andrews TJ Conroy JP von Caemmerer S 2005 Faster Rubisco is the key to superior nitrogen-use efficiency in NADP-malic enzyme relative to NAD-malic enzyme C(4) grasses. Plant Physiology 137, 638–650. 15665246 10.1104/pp.104.054759 PMC1065364

[CIT0014] Giordano M Beardall J Raven JA 2005 CO_2_ concentrating mechanisms in algae: mechanisms, environmental modulation, and evolution. Annual Review of Plant Biology 56, 99–131. 10.1146/annurev.arplant.56.032604.144052 15862091

[CIT0015] Haslam RP Keys AJ Andralojc PJ Madgwick PJ Andersson I Grimsrud A Eilertsen HC Parry MAJ 2005 Specificity of diatom Rubisco. In: Omasa K Nouchi I De Kok LJ, eds. Plant responses to air pollution and global change. Springer: Japan, 157–164.

[CIT0016] Hauser T Popilka L Hartl FU Hayer-Hartl M 2015 Role of auxiliary proteins in Rubisco biogenesis and function. Nature Plants 1, 15065. 27250005 10.1038/nplants.2015.65

[CIT0017] Hennon GMM Ashowrth J Groussman RD Berthiaume C Morales RL Baliga NS Orellana MW Armbrust EV 2015 Diatom acclimation to elevated CO_2_ via novel gene clusters and cAMP-signaling. Nature Climate Change 5, 761–765.

[CIT0018] Heureux AMC Rickaby REM 2015 Refining our estimate of atmospheric CO_2_ across the Eocene–Oligocene climatic transition. Earth and Planetary Science Letters 409, 329–338.

[CIT0019] Hopkinson B 2014 A chloroplast pump model for the CO_2_ concentrating mechanism in the diatom *Phaeodactylum tricornutum* . Photosynthesis Research 121, 223–233. 24292858 10.1007/s11120-013-9954-7

[CIT0020] Hopkinson BM Dupont CL Allen AE Morel FMM 2011 Efficiency of the CO_2_-concentrating mechanism of diatoms. Proceedings of the National Academy of Sciences, USA 108, 3830–3837. 10.1073/pnas.1018062108 PMC3054024 21321195

[CIT0021] Hopkinson BM Young JN Tansik AL Binder BJ 2014 The minimal CO_2_-concentrating mechanism of *Prochlorococcus* spp. MED4 is effective and efficient. Plant Physiology 166, 2205–2217. 25315602 10.1104/pp.114.247049 PMC4256842

[CIT0022] Kane HJ Viil J Entsch B Paul K Morell MK Andrews TJ 1994 An improved method for measuring the CO_2_/O_2_ specificity of ribulose-bisphosphate carboxylase-oxygenase. Australian Journal of Plant Physiology 21, 449–461.

[CIT0023] Kane HJ Wilkin J-M Portis AR John Andrews T 1998 Potent inhibition of ribulose-bisphosphate carboxylase by an oxidized impurity in ribulose-1,5-bisphosphate. Plant Physiology 117, 1059–1069. 9662549 10.1104/pp.117.3.1059 PMC34922

[CIT0024] Kranz SA Young JN Hopkinson B Goldman JAL Tortell PD Morel FMM 2015 Low temperature reduces the energetic requirement for the CO_2_ concentrating mechanism in diatoms. New Phytologist 205, 192–202. 25308897 10.1111/nph.12976

[CIT0025] Kroth PG 2015 The biodiversity of carbon assimilation. Plant Physiology 172, 76–81. 10.1016/j.jplph.2014.07.021 25239594

[CIT0026] Laing WA Ogren WL Hageman RH 1974 Regulation of soybean net photosynthetic CO_2_ fixation by the interaction of CO_2_, O_2_, and ribulose 1,5-diphosphate carboxylase. Plant Physiology 54, 678–685. 16658951 10.1104/pp.54.5.678 PMC366581

[CIT0027] Long BM Bahar NHA Atkin OK 2015 Contributions of photosynthetic and non-photosynthetic cell types to leaf respiration in *Vicia faba L*. and their responses to growth temperature. Plant, Cell and Environment 38, 2263–2276. 10.1111/pce.12544 25828647

[CIT0028] Long SP 1999 Environmental responses. In: Sage RF Monson RK, eds. C_4_ plant biology. Academic Press: San Diego, 215–249.

[CIT0029] Losh JL Young JN Morel FM 2013 Rubisco is a small fraction of total protein in marine phytoplankton. New Phytologist 198, 52–58. 23343368 10.1111/nph.12143

[CIT0030] Mueller-Cajar O Stotz M Bracher A 2014 Maintaining photosynthetic CO_2_ fixation via protein remodelling: the Rubisco activases. Photosynthesis Research 119, 191–201. 23543331 10.1007/s11120-013-9819-0

[CIT0031] Mueller-Cajar O Whitney S 2008 Directing the evolution of Rubisco and Rubisco activase: first impressions of a new tool for photosynthesis research. Photosynthesis Research 98, 667–675. 18626786 10.1007/s11120-008-9324-z PMC2758363

[CIT0032] Nakajima K Tanaka A Matsuda Y 2013 SLC4 family transporters in a marine diatom directly pump bicarbonate from seawater. Proceedings of the National Academy of Sciences, USA 110, 1767–1772. 10.1073/pnas.1216234110 PMC3562803 23297242

[CIT0033] Nelson DM Tréguer P Brzezinski MA Leynaert A Quéguiner B 1995 Production and dissolution of biogenic silica in the ocean: revised global estimates, comparison with regional data and relationship to biogenic sedimentation. Global Biogeochemical Cycles 9, 359–372.

[CIT0034] Osborne CP Sack L 2012 Evolution of C_4_ plants: a new hypothesis for an interaction of CO_2_ and water relations mediated by plant hydraulics. Philosophical Transactions of the Royal Society B: Biological Sciences 367, 583–600. 10.1098/rstb.2011.0261 PMC3248710 22232769

[CIT0035] Parry MAJ Andralojc PJ Scales JC Salvucci ME Carmo-Silva AE Alonso H Whitney SM 2013 Rubisco activity and regulation as targets for crop improvement. Journal of Experimental Botany 64, 717–730. 23162118 10.1093/jxb/ers336

[CIT0036] Peterhansel C Niessen M Kebeish RM 2008 Metabolic engineering towards the enhancement of photosynthesis. Photochemistry and Photobiology 84, 1317–1323. 18764897 10.1111/j.1751-1097.2008.00427.x

[CIT0037] Pierce J Tolbert NE Barker R 1980 Interaction of ribulose-bisphosphate carboxylase/oxygenase with transition-state analogues. Biochemistry 19, 934–942. 7356969 10.1021/bi00546a018

[CIT0038] Price GD Badger MR Woodger FJ Long BM 2008 Advances in understanding the cyanobacterial CO_2_-concentrating-mechanism (CCM): functional components, Ci transporters, diversity, genetic regulation and prospects for engineering into plants. Journal of Experimental Botany 59, 1441–1461. 17578868 10.1093/jxb/erm112

[CIT0039] Price GD Evans JR von Caemmerer S Yu J-W Badger MR 1995 Specific reduction of chloroplast glyceraldehyde-3-phosphate dehydrogenase activity by antisense RNA reduces CO_2_ assimilation via a reduction in ribulose bisphosphate regeneration in transgenic tobacco plants. Planta 195, 369–378. 7766043 10.1007/BF00202594

[CIT0040] Raven J Beardall J Giordano M 2014 Energy costs of carbon dioxide concentrating mechanisms in aquatic organisms. Photosynthesis Research 121, 111–124. 24390639 10.1007/s11120-013-9962-7

[CIT0041] Raven J Giordano M Beardall J Maberly SC 2011 Algal and aquatic plant carbon concentrating mechanisms in relation to environmental change. Photosynthesis Research 109, 1–16. 10.1007/s11120-011-9632-6 21327536

[CIT0042] Raven JA 2009 Contributions of anyoxygenic and oxygenic phototrophy and chemolithotrophy to carbon and oxygen fluxes in aquatic environments. Aquatic Microbial Ecology 56, 177–192.

[CIT0043] Raven JA Beardall J 2016 The ins and outs of CO_2_ . Journal of Experimental Botany 67, 1–13. 26466660 10.1093/jxb/erv451 PMC4682431

[CIT0044] Raven JA Giordano M Beardall J Maberly SC 2012 Algal evolution in relation to atmospheric CO_2_: carboxylases, carbon-concentrating mechanisms and carbon oxidation cycles. Philosophical Transactions of the Royal Society B: Biological Sciences 367, 493–507. 10.1098/rstb.2011.0212 PMC3248706 22232762

[CIT0045] Read BA Tabita FR 1994 High substrate specificity factor ribulose bisphosphate carboxylase/oxygenase from eukaryotic marine algae and properties of recombinant cyanobacterial rubisco containing ‘algal’ residue modifications. Archives of Biochemistry and Biophysics 312, 210–218. 8031129 10.1006/abbi.1994.1301

[CIT0046] Reinfelder JR 2010 Carbon concentrating mechanisms in eukaryotic marine phytoplankton. Annual Review of Marine Science 3, 291–315. 10.1146/annurev-marine-120709-142720 21329207

[CIT0047] Reinfelder JR Kraepiel AM Morel FM 2000 Unicellular C_4_ photosynthesis in a marine diatom. Nature 407, 996–999. 11069177 10.1038/35039612

[CIT0048] Reinfelder JR Milligan AJ Morel FMM 2004 The role of the C_4_ pathway in carbon accumulation and fixation in a marine diatom. Plant Physiology 135, 2106–2111. 15286292 10.1104/pp.104.041319 PMC520782

[CIT0049] Rigobello-Masini M Penteado JCP Tiba M Masini JC 2012 Study of photorespiration in marine microalgae through the determination of glycolic acid using hydrophilic interaction liquid chromatography. Journal of Separation Science 35, 20–28. 22128110 10.1002/jssc.201100488

[CIT0050] Roberts K Granum E Leegood RC Raven JA 2007 Carbon acquisition by diatoms. Photosynthesis Research 93, 79–88. 17497225 10.1007/s11120-007-9172-2

[CIT0051] Rost B Riebesell U Burkhardt S Sültemeyer D 2003 Carbon acquisition of bloom-forming marine phytoplankton. Limnology and Oceanography 48, 55–67.

[CIT0053] Sage RF 2001 Environmental and evolutionary preconditions for the origin and diversification of the C_4_ photosynthetic syndrome. Plant Biology 3, 202–213.

[CIT0054] Sage RF 2004 Tansley review: the evolution of C_4_ photosynthesis. New Phytologist 161, 341–371. 10.1111/j.1469-8137.2004.00974.x 33873498

[CIT0055] Savir Y Noor E Milo R Tlusty T 2010 Cross-species analysis traces adaptation of Rubisco toward optimality in a low-dimensional landscape. Proceedings of the National Academy of Sciences, USA 107, 3475–3480. 10.1073/pnas.0911663107 PMC2840432 20142476

[CIT0056] Schnitzler Parker M Armbrust E Piovia-Scott J Keil RG 2004 Induction of photorespiration by light in the centric diatom *Thalassiosisra weissflogii* (Bacillariophyceae): molecular characterization and physiological consequences. Journal of Phycology 40, 557–567.

[CIT0057] Seemann JR Badger MR Berry JA 1984 Variations in the specific activity of ribulose-1,5-bisphosphate carboxylase between species utilizing differing photosynthetic pathways. Plant Physiology 74, 791–795. 16663511 10.1104/pp.74.4.791 PMC1066769

[CIT0058] Sharwood RE von Caemmerer S Maliga P Whitney SM 2008 The catalytic properties of hybrid rubisco comprising tobacco small and sunflower large subunits mirror the kinetically equivalent source rubiscos and can support tobacco growth. Plant Physiology 146, 83–96. 17993544 10.1104/pp.107.109058 PMC2230571

[CIT0059] Sunda WG Price NM Morel FMM 2005 Trace metal ion buffers and their use in culture studies. In: Andersen RA, ed. Algal culturing techniques. Amsterdam: Elsevier, 35–63.

[CIT0060] Tabita FR Satagopan S Hanson TE Kreel NE Scott SS 2008 Distinct form I, II, III, and IV Rubisco proteins from the three kingdoms of life provide clues about Rubisco evolution and structure/function relationships. Journal of Experimental Botany 59, 1515–1524. 18281717 10.1093/jxb/erm361

[CIT0061] Tcherkez G 2013 Modelling the reaction mechanism of ribulose-1,5-bisphosphate carboxylase/oxygenase and consequences for kinetic parameters. Plant, Cell and Environment 36, 1586–1596. 10.1111/pce.12066 23305122

[CIT0062] Tcherkez G 2015 The mechanism of Rubisco-catalyzed oxygenation. Plant, Cell and Environment 39 (5), 983–997. 10.1111/pce.12629 26286702

[CIT0063] Tcherkez GGB Farquhar GD Andrews TJ 2006 Despite slow catalysis and confused substrate specificity, all ribulose bisphosphate carboxylases may be nearly perfectly optimized. Proceedings of the National Academy of Sciences, USA 103, 7246–7251. 10.1073/pnas.0600605103 PMC1464328 16641091

[CIT0064] Tortell PD Rau GH Morel FM 2000 Inorganic carbon acquisition in coastal Pacific phytoplankton communities. Limnology and Oceanography 45, 1485–1500.

[CIT0065] Tsai YC Lapina MC Bhushan S Mueller-Cajar O 2016 Identification and characterization of multiple rubisco activases in chemoautotrophic bacteria. Nature Communications. 6, 8883. 10.1038/ncomms9883 PMC4660213 26567524

[CIT0066] von Caemmerer S Furbank R 2003 The C_4_ pathway: an efficient CO_2_ pump. Photosynthesis Research 77, 191–207. 16228376 10.1023/A:1025830019591

[CIT0067] Way DA Katul GG Manzoni S Vico G 2014 Increasing water use efficiency along the C_3_ to C_4_ evolutionary pathway: a stomatal optimization perspective. Journal of Experimental Botany 65, 3683–3693. 24860185 10.1093/jxb/eru205 PMC4085968

[CIT0068] Whitehead L Long BM Price GD Badger MR 2014 Comparing the in vivo function of α-carboxysomes and β-carboxysomes in two model cyanobacteria. Plant Physiology 165, 398–411. 24642960 10.1104/pp.114.237941 PMC4012598

[CIT0069] Whitney SM Baldet P Hudson GS Andrews TJ 2001 Form I Rubiscos from non-green algae are expressed abundantly but not assembled in tobacco chloroplasts. The Plant Journal 26, 535–547. 11439139 10.1046/j.1365-313x.2001.01056.x

[CIT0070] Whitney SM Houtz RL Alonso H 2011 Advancing our understanding and capacity to engineer nature’s CO_2_-sequestering enzyme, Rubisco. Plant Physiology 155, 27–35. 20974895 10.1104/pp.110.164814 PMC3075749

[CIT0071] Whitney SM Sharwood RE 2007 Linked Rubisco subunits can assemble into functional oligomers without impeding catalytic performance. Journal of Biological Chemistry 282, 3809–3818. 17150955 10.1074/jbc.M610479200

[CIT0072] Whitney SM von Caemmerer S Hudson GS Andrews TJ 1999 Directed mutation of the Rubisco large subunit of tobacco influences photorespiration and growth. Plant Physiology 121, 579–588. 10517850 10.1104/pp.121.2.579 PMC59421

[CIT0073] Yeoh H-H Badger MR Watson L 1980 Variations in K_m_(CO_2_) of ribulose-1,5-bisphosphate carboxylase among grasses. Plant Physiology 66, 1110–1112. 16661586 10.1104/pp.66.6.1110 PMC440799

[CIT0074] Yoon HS Hackett JD Pinto G Bhattacharya D 2002 The single, ancient origin of chromist plastids. Proceedings of the National Academy of Sciences, USA 99, 15507–15512. 10.1073/pnas.242379899 PMC137747 12438651

[CIT0075] Young JN Bruggeman J Rickaby REM Erez J Conte M 2013 Evidence for changes in carbon isotopic fractionation by phytoplankton between 1960 and 2010. Global Biogeochemical Cycles 27, 505–515.

[CIT0076] Young JN Kranz SA Goldman JAL Tortell PD Morel FM 2015 Antarctic phytoplankton down-regulate their carbon concentrating mechanisms under high CO_2_ with no change in growth rates. Marine Ecology Progress Series 532, 13–28.

[CIT0077] Young JN Rickaby REM Kapralov MV Filatov DA 2012 Adaptive signals in algal Rubisco reveal a hisotry of ancient atmospheric CO_2_ . Philosophical Transactions of the Royal Society B: Biological Sciences 367, 483–492. 10.1098/rstb.2011.0145 PMC3248704 22232761

[CIT0078] Zelitch I Schultes NP Peterson RB Brown P Brutnell TP 2009 High glycolate oxidase activity is required for survival of maize in normal air. Plant Physiology 149, 195–204. 18805949 10.1104/pp.108.128439 PMC2613714

[CIT0079] Zhu G Jensen RG 1991 Xyulose 1,5-bisphosphate synthesized by ribulose 1,5-bisphosphate carboxylase/oxygenase during catalysis binds to decarbamylated enzyme. Plant Physiology 97, 1348–1353. 16668555 10.1104/pp.97.4.1348 PMC1081170

