# Supplementary material for: Large variation in the Rubisco kinetics of diatoms reveals diversity among their carbon-concentrating mechanisms
Source: J Exp Bot. 2016 Apr 29;67(11):3445–56. doi: 10.1093/jxb/erw163 (PMC4892730; doi:10.1093/jxb/erw163)
Supplement: Supplementary Data [file supp_67_11_3445__index.html]

Large variation in the Rubisco kinetics of diatoms reveals diversity among their carbon-concentrating mechanisms — Large variation in the Rubisco kinetics of diatoms reveals diversity among their carbon-concentrating mechanisms — Supplementary Data 

# Large variation in the Rubisco kinetics of diatoms reveals diversity among their carbon-concentrating mechanisms

## Supplementary Data

Data files

- supplementary\_table\_S1.pdf - Supplementary Data
