# Supplementary material for: Large variation in the Rubisco kinetics of diatoms reveals diversity among their carbon-concentrating mechanisms
Source: J Exp Bot. 2016 Apr 29;67(11):3445–56. doi: 10.1093/jxb/erw163 (PMC4892730; doi:10.1093/jxb/erw163)
Supplement: Supplementary Data [file supp_erw163_supplementary_table_S1.pdf]

Large variation in the Rubisco kinetics of diatoms reveals diversity among their carbon concentrating mechanisms.

YN Jodi, AMC Heureux, RE Sharwood, REM Rickaby, FMM Morel, and S Whitney

Supplementary Table S1 Rubisco kinetics at 25 ºC taken from other datasets and used in Figure 2

	k_{cat}^{c} (s^{-1})	K_C (µM)	K_O (µM)	Sc/o ($mol.mol^{-1}$)	calc k_{cat}^{o} (s^{-1})	Dataset reference
**Red Algae**						
Griffithsia monilis	2.6	9	467	167	0.78	Savir *et al*. (2010)
Galdiera sulfuraria	1.2	3	374	166	0.82	Savir *et al*. (2010)
Porphyridium cruentum	1.6	22	1574	129	0.89	Badger *et al*. (1998)
Cyanidium caldarium	1.3	7	n.d.	224	n.d.	Badger *et al*. (1998)
Cyanidium partita	1.6	7	n.d.	238	n.d.	Badger *et al*. (1998)
**Green algae**						
Scenedesmus obliquus	n.d.	38	660	63	n.d.	Savir *et al*. (2010)
Chlamydomonas reinhardtii	5.8	29	480	61	1.57	Savir *et al*. (2010)
Euglena gracilis	n.d.	25	410	54	n.d.	Savir *et al*. (2010)
**Bryophytes**						Galmes *et al*. (2014)
Atrichum undulatum	3.0	19	806	n.d.	n.d.	Galmes *et al*. (2014)
Marchantia polymorpha	2.6	13	565	n.d.	n.d.	Galmes *et al*. (2014)
**Ferns**						
Pteridium aquilinum	4.0	14	391	n.d.	n.d.	Galmes *et al*. (2014)
Platycerium superbum	2.3	12	459	n.d.	n.d.	Galmes *et al*. (2014)
**Gymnosperms**						
Cycas panzhihuaensis	3.1	9	525	n.d.	n.d.	Galmes *et al*. (2014)
Metasequoia glyptostroboides	2.7	16	561	n.d.	n.d.	Galmes *et al*. (2014)
Basal angiosperm						
Nymphaea alba	2.4	11	438	n.d.	n.d.	Galmes *et al*. (2014)
**Aquatic macrophyte using HCO_3^-**						Galmes *et al*. (2014)
Ceratophyllum demersum	3.5	14	317	n.d.	n.d.	Galmes *et al*. (2014)
**CAM plants**						
Agave victoriae–reginae	3.4	10	400	n.d.	n.d.	Galmes *et al*. (2014)
Carpobrotus edulis	3.6	10	313	n.d.	n.d.	Galmes *et al*. (2014)
Echeveria elegans	4.5	11	236	n.d.	n.d.	Galmes *et al*. (2014)
**Carnivorous plants**						
Drosera capensis	4.7	10	327	n.d.	n.d.	Galmes *et al*. (2014)
Drosera venusta	3.3	12	348	n.d.	n.d.	Galmes *et al*. (2014)
Sarracenia flava	4.4	13	426	n.d.	n.d.	Galmes *et al*. (2014)
**C_3**						
Arabidopsis thaliana	4.1	10	333	n.d.	n.d.	Galmes *et al*. (2014)
Chenopodium alba	2.9	11	415	79	1.37	Savir *et al*. (2010)
Crithmum maritimum	3.4	9	183	n.d.	n.d.	Galmes *et al*. (2014)
Dactylis glomerata cv. Porto	3.2	11	453	n.d.	n.d.	Galmes *et al*. (2014)
Eucalyptus moorei	3.2	10	285	n.d.	n.d.	Galmes *et al*. (2014)
Eucalyptus neglecta	2.5	8	230	n.d.	n.d.	Galmes *et al*. (2014)
Flaveria pringlei	3.1	12	666	81	2.12	Savir *et al*. (2010)
Glycine max	n.d.	9	430	82	n.d.	Savir *et al*. (2010)
Iris douglasiana	3.5	10	413	n.d.	n.d.	Galmes *et al*. (2014)
Limonium latebracteatum	2.7	9	344	n.d.	n.d.	Galmes *et al*. (2014)
Limonium stenophyllum	2.6	8	457	n.d.	n.d.	Galmes *et al*. (2014)
Limonium virgatum	2.4	9	381	n.d.	n.d.	Galmes *et al*. (2014)
lolium perenne	n.d.	16	500	80	n.d.	Galmes *et al*. (2014)
Nicotiana tabacum	3.4	11	295	82	1.14	Galmes *et al*. (2014)
Nicotiana tabacum (this)	3.1	10	283	83	1.09	this study
Pallenis maritima	2.7	6	321	n.d.	n.d.	Galmes *et al*. (2014)
Sideritis cretica subsp. spicata	2.0	8	328	n.d.	n.d.	Galmes *et al*. (2014)
Spnacia oleracea	3.7	14	480	80	1.59	Savir *et al*. (2010)
Tetragonium expnasa	n.d.	13	600	81	n.d.	Savir *et al*. (2010)
Teucrium heterophyllum	2.7	7	359	n.d.	n.d.	Galmes *et al*. (2014)

Trachycarpus fortunei	2.8	9	364	n.d.	n.d.	Galmes *et al.* (2014)
Triticum aestivum	2.5	14	730	90	1.45	Savir *et al.* (2010)
Triticum aestivum cv. Alexandria	4.0	10	414	n.d.	n.d.	Galmes *et al.* (2014)
**C_4**						
Amaranthus edulis	4.1	18	289	77	0.85	Whitney *et al.* (2011), Savir *et al.* (2010)
Amaranthus hybridus	3.8	16	640	82	1.85	Whitney *et al.* (2011), Savir *et al.* (2010)
Cynodon dactylon	3.7	21	n.d.	89	n.d.	Whitney *et al* (2011)
Flaveria australasica	3.8	22	309	77	0.69	Whitney *et al.* (2011), Savir *et al.* (2010)
Flaveria bidentis	4.2	20.2	n.d.	76	n.d.	Whitney *et al.* (2011)
Flaveria kochiana	3.7	22.7	n.d.	77	n.d.	Whitney *et al.* (2011)
Flaveria trinervia	4.4	17.9	n.d.	77	n.d.	Whitney *et al.* (2011)
Paspalum dilatatum	3.4	19.9	n.d.	88	n.d.	Whitney *et al.* (2011)
Potuloca oleraca	5.9	14	n.d.	78	n.d.	Savir *et al.* (2010)
Sorghum bicolor	5.4	30	n.d.	70	n.d.	Whitney *et al.* (2011)
Sorghum bicoor	5.4	30	n.d.	70	n.d.	Savir *et al.* (2010)
Zea mays	4.4	34	810	78	1.34	Savir *et al.* (2010)
Zea mays (this study)	5.5	19	397	88	1.31	this study
Zoysia japonica	4.1	18.5	n.d.	84	n.d.	Whitney *et al.* (2011)

* also used in the analysis of Figure 1

References

Badger MRT, Andrews J, Whitney SM, Ludwig M, Yellowlees DC, Leggat W, Price GD. (1998) The diversity and coevolution of Rubisco, plastids, pyrenoids, and chloroplast-based CO_2-concentrating mechanisms in algae. Canadian Journal of Botany 76: 1052-1071

Galmes J, Kapralov MV, Andralojc PJ, Conesa MÀ, Keys AJ, Parry MAJ, Flexas J. (2014) Expanding knowledge of the Rubisco kinetics variability in plant species: environmental and evolutionary trends. Plant, Cell & Environment **37:** 1989-2001

Savir Y, Noor E, Milo R, Tlusty T. (2010) Cross-species analysis traces adaptation of Rubisco toward optimality in a low-dimensional landscape. Proceedings of the National Academy of Sciences 107: 3475-3480

Whitney SM, Houtz RL, Alonso H. (2011) Advancing Our Understanding and Capacity to Engineer Nature's CO_2-Sequestering Enzyme, Rubisco. Plant Physiology 155: 27-35
